# Antiviral activity of cathelicidins against porcine epidemic diarrhea virus (PEDV): Mechanisms, and efficacy

**DOI:** 10.1016/j.virusres.2024.199496

**Published:** 2024-11-15

**Authors:** Fatemeh Pashaie, Tabitha E. Hoornweg, Floris J. Bikker, Tineke Veenendaal, Femke Broere, Edwin J.A. Veldhuizen

**Affiliations:** a Department of Biomolecular Health Sciences, Division Infectious Diseases & Immunology, Faculty of Veterinary Medicine, Utrecht University, Utrecht 3584 CL, the Netherlands; b Department of Oral Biochemistry, Academic Centre for Dentistry Amsterdam, University of Amsterdam and VU University Amsterdam, Amsterdam 1081 LA, the Netherlands; c Cell Microscopy Core, Center for Molecular Medicine, University Medical Center Utrecht, Utrecht 3584CX, the Netherlands

**Keywords:** Antimicrobial peptides, Cathelicidins, Antiviral activity, Porcine epidemic diarrhea virus, Vero cells

## Abstract

Porcine epidemic diarrhea virus (PEDV) is a harmful coronavirus infecting pigs, which is resulting in substantial financial losses in the global pig industry. The lack of effective vaccines or treatments underscores the pressing need for new antiviral strategies. Antimicrobial peptides (AMPs), specifically cathelicidins such as LL-37, have demonstrated promising activity against a range of viruses. This study aims to elucidate the antiviral mechanisms of cathelicidins by examining their inhibitory capabilities against PEDV *in vitro*. Four pig-derived antimicrobial peptides (PMAP-36, PMAP-23, PR-39, and PG-1), together with chicken-derived CATH-B1 and human-derived LL-37 were analyzed for their anti-PEDV activity. Flow cytometry and fluorescent microscopy confirmed that LL-37 and CATH-B1 had strong inhibitory effects at non-toxic concentrations of 5 and 10 µM, significantly reducing GFP-PEDV infection of Vero cells both in co- and pre-incubation setups. In contrast, none of the porcine peptides exhibited any inhibitory effects, even at higher doses. Fluorogenic LL-37 was shown to enter VERO cells, indicative of a possible immunomodulatory antiviral mode of action. However, transmission electron microscopy clearly indicated that both LL-37 and CATH-B1 affected virus morphology and caused aggregation of viral particles, showing that peptide-virus interaction caused reduced virus infectivity. In conclusion, this analysis highlights the potential of LL-37 and CATH-B1 as inhibitors against PEDV, suggesting promising directions for innovative therapeutic antiviral strategies.

## Highlights

• A flow cytometry based method to detect anti-PEDV activity was set up.
• 2 antimicrobial peptides were actively repressing PEDV infectivity.
• LL-37 entered VERO- cells and prevented subsequent PEDV infection.
• TEM showed direct effects of LL-37 and CATH-B1 on virus morphology.

## 1 Introduction

Porcine epidemic diarrhea virus (PEDV) is an enveloped single-stranded RNA virus, classified under the Alphacoronavirus genus in the Coronaviridae family and the Nidovirales order. It is pathogenic to swine of all ages, causing symptoms like anorexia, vomiting, watery diarrhea, dehydration, and causes high mortality (Kirchdoerfer et al., 2021; Z Li et al., 2020; Lin et al., 2022). PEDV outbreaks have had a pronounced economic impact on the swine industry, rendering it a formidable hazard to the industry worldwide. Notably, the 2013–2014 PEDV outbreak in the United States resulted in considerable financial repercussions, with losses estimated to range between $900 million and $1.8 billion (Yang et al., 2019; Chen et al., 2020; Langel et al., 2016). PEDV primarily targets porcine intestinal epithelial cells, leading to subsequent extensive infection of the intestinal tract, which triggers processes of atrophy and necrosis within the structure of the intestinal villi. Moreover, infection significantly impairs the absorption of vital nutrients, consequently eliciting a spectrum of clinical manifestations including emetic episodes, diarrheal incidents, notable reduction in body mass, decreased appetite, and prevailing despondency. The excretion of PEDV into the external milieu via fecal discharge further precipitates the potential for a widespread epidemic event upon contamination of the surrounding environment (Z Li et al., 2020). According to reports, neonatal pigs are more susceptible to PEDV compared to older pigs due to several factors including a slower regeneration of enterocytes, and an anatomically and physiologically immature large intestine (Jung et al., 2020).

The first detection of PEDV in swine dates back to the late 1970s, with its initial identification in the United Kingdom in 1977 and Belgium in 1978. Nonetheless, the emergence of novel variants of PEDV has occurred more recently, as evidenced by the 2011 outbreak in China, which was followed by subsequent occurrences in the Americas, Europe, and Asia (Lin et al., 2022; Yang et al., 2019). Despite the development of various vaccines for PEDV, their effectiveness has been limited. For example, the initial success of vaccine CV777, which was employed in 1990, was compromised by the emergence of a new strain of PEDV in 2011. Consequently, the pursuit of antiviral drugs has emerged as a realistic approach to control the spread of the virus (Chen et al., 2020; X Li et al., 2020). The development of antiviral drugs began with the approval of idoxuridine in the 1960s, marking the onset of a field that has seen over 90 human antiviral drugs approved in the last six decades. These drugs can be broadly categorized into two groups: virus-targeting and host-directed antivirals. Virus-targeting antivirals operate through direct or indirect inhibition of viral proteins' biological activity or disruption of the viral particle structure. In contrast, host-directed antivirals function by obstructing host factors that viruses exploit during their life cycle (Zakaryan et al., 2021).

Antimicrobial peptides (AMPs) represent a promising class of antiviral compounds. They comprise a diverse family of peptides with various functions, including direct antimicrobial activity against a range of viral, bacterial, and fungal pathogens. Their recently described immunomodulatory functions are particularly noteworthy, as these provide further potential for clinical applications. Among AMPs, the cathelicidin family has attracted a lot of interest due to its well-studied immunomodulatory properties (Scheenstra et al., 2020). Cathelicidins exhibit significant structural diversity across species, ranging from proline-rich structures to amphipathic α-helices as well as β-sheet containing peptides. These peptides have been identified in a diverse array of animals, including mammals, birds, reptiles, fish, and amphibians. In contrast to humans, rabbits, and mice that express a single cathelicidin type, other species such as pigs, cows, and chickens have multiple and more structurally diverse cathelicidins (Zakaryan et al., 2021; Wieczorek et al., 2010).

LL-37, as the sole human cathelicidin, possesses the ability to inhibit viruses such as dengue virus (DENV), human immunodeficiency virus (HIV), respiratory syncytial virus (RSV), human rhinovirus (HRV), vaccinia virus (VACV), herpes simplex virus (HSV), zika virus (ZIKV), hepatitis C virus (HCV), and venezuelan equine encephalitis virus (VEEV) (A Ahmed et al., 2019; Harcourt et al., 2016; Findlay et al., 2016; Matsumura et al., 2016; Alagarasu et al., 2017; Tripathi et al., 2015). This inhibition is achieved through multiple mechanisms, including direct interaction with virions, enhancement of type I interferon (IFN) expression, and suppression of pro-inflammatory cytokine production (A Ahmed et al., 2019; Harcourt et al., 2016; Findlay et al., 2016; Matsumura et al., 2016; Alagarasu et al., 2017; Tripathi et al., 2015). The cathelicidin CATH-B1, originally found in chickens, demonstrates antiviral activity by effectively inhibiting the entry of influenza A virus (IAV). This inhibition occurs through viral agglutination, thereby hindering the virus's capacity for invasion (Peng et al., 2020). Although there have been several *in vitro* studies investigating the antiviral properties of LL-37 and CATH-B1, the precise mechanism underlying their antiviral activity remains unclear to date.

Numerous research studies have extensively investigated the antibacterial properties of cathelicidins (Nagaoka et al., 2020; Lee et al., 2016; Blodkamp et al., 2016; J Chen et al., 2021). However, there is no consensus on the exact antiviral mechanism of action of cathelicidins and it is likely that it can differ for each virus and cathelicidins combination. Consequently, this study aimed to address the inhibitory abilities and antiviral mechanisms of several cathelicidins against PEDV *in vitro*. The findings of this study will provide valuable insights into the antiviral mechanisms of cathelicidins and their potential application as effective antiviral drugs.

## 2 Materials and methods

### 2.1 Antimicrobial peptides

Peptides were synthesized by China peptides (Shanghai, China) and the Academic Centre for Dentistry Amsterdam (ACTA, The Netherlands) using Fmoc-chemistry (CPC Scientific, Sunnyvale, CA, USA). All peptides were purified by reverse-phase high-performance liquid chromatography to a purity of >95%. Protegrin-1 was a kind gift of Thomas Wood and Nathaniel Martin (Molecular Biotechnology, Institute of Biology, Leiden University, the Netherlands, Leiden). The peptides were dissolved in water at a concentration of 1600 µM, aliquoted, and stored at −80 °C until required for subsequent experiments.

Table 1

**Table 1:** Characteristics of the main cathelicidins utilized in this study.

AMPs	Species	Amino acid sequence	Length	Charge
LL-37	Human	LLGDFFRKSKEKIGKEFKRIVQRIKDFLRNLVPRTES	37	+6
Fluorogenic LL-37	Human	Tamra-(ahx)-LLGDFFRKSKEKIGKEFKRIVQRIKDFLRNLVPRTES	37	+6
CATH-B1	Chicken	PIRNWWIRIWEWLNGIRKRLRQRSPFYVRG HLNVTSTPQP	40	+7
PMAP-36	Porcine	GRFRRLRKKTRKRLKKIGKVLKWIPPIVGSIPLGCG	36	+13
PMAP-23	Porcine	RIIDLLWRVRRPQKPKFVTVWVR	23	+6
PR-39	Porcine	RRRPRPPYLPRPRPPPFFPPRLPPRIPPGFPPRFPPRFP	39	+10
PG-1	Porcine	RGGRLCYCRRRFCVCVGR	18	+6

### 2.2 Cell culture

Vero-mCeacam cells (Vero cells), derived from African green monkey kidney cells, were maintained in DMEM (Gibco, Thermo Fisher Scientific, Waltham, MA) supplemented with 10% FCS (Bodinco B.V., Alkmaar, The Netherlands) and 200 units/mL penicillin, and 200 mg/mL streptomycin (P/S) (Gibco, Life Technologies Limited, Paisley, UK) at 37 °C and 5% CO_2_.

### 2.3 PEDV cultivation

PEDV and PEDV-GFP were a kind gift of Berend Jan Bosch (Department of Biomolecular Health Sciences, Faculty of Veterinary Medicine, Utrecht University, The Netherlands) and preserved and propagated using Vero cells as described before (Li et al., 2013). To prepare viral stocks, Vero cells were seeded in a T75 flask and cultured in DMEM complete medium supplemented with 10% FCS at 37 °C and 5% CO_2_, until reaching 90% confluency. Subsequently, the Vero cells were exposed to PEDV by incubating them with a 100 µL viral solution in 1900 µL of PBS at 37 °C for 2 h Following this, the flask was supplemented with 10 mL of DMEM complete medium and incubated until cytopathic effects were observed using light microscopy at 96 h post-infection. To release the virus from the Vero cells, the culture flask was repeatedly frozen at −20 °C and then thawed at room temperature (RT) (4 times). The supernatant was subsequently collected into a 15 mL centrifuge tube. Cell debris was eliminated by centrifugation at 350 × *g* for 5 min, and the resulting supernatant was aliquoted and stored at −80 °C.

### 2.4 Virus titration

Plaque assays were performed by culturing Vero cells at a density of 24 × 10^5^ cells per well in 6-well plates. The cells were infected with 10-fold serial dilutions of virus and incubated at 37 °C for 2 h After removing the virus inoculum, cells were overlaid with 1 mL overlay solution, consisting of equal volumes of 1% Agarose (Sea Plaque GTG agarose; Lonza) and 1x MEM (Gibco, Temin's modification (2X), without phenol red, NY, USA), and incubated at 37 °C for 5 days with 1 mL of overlay medium. Following fixation with 0.5 mL of 10% paraformaldehyde (PFA) (Sigma-Aldrich, P6148, Darmstadt, Germany), plates were stained with crystal violet (0.1% crystal violet (Abcam, Germany) in 20% methanol). Subsequently, plaques were counted to determine the viral titer (Fig. S1).

### 2.5 Cell viability assay

Vero cells were seeded at a density of 1 × 10^5^ cells per well in 96-well plates. The cells were then stimulated with 100 µL of AMPs (0–10 µM) in DMEM for either 3 or 24 h Subsequently, the culture medium was replaced with 100 μL of medium containing 10% water-soluble tetrazolium 1 (WST-1) (Roche, Basel, Switzerland). After 10–15 min of incubation, the colorimetric changes were measured at 450 nm using a FLUOstar Omega microplate reader (BMG Labtech GmbH, Ortenberg, Germany). Metabolic activity was calculated relative to the control group without AMPs.

### 2.6 Lactate dehydrogenase (LDH) assay

To assess the cytotoxicity of peptides, Vero cells were treated with AMPs (0 − 10 μM in DMEM) as outlined in paragraph 2.5. After 3 and 24 h of stimulation, the fraction of lactate dehydrogenase (LDH) released into the culture supernatant was measured using the Cyto Tox 96 nonradioactive cytotoxicity kit (Promega GmbH, Walldorf, Germany) according to the manufacturer's instructions, and expressed as a percentage of the maximum LDH release from cells, lysed by 1% Triton X-100 (Sigma-Aldrich, T8787, Darmstadt, Germany).

### 2.7 *In vitro* antiviral activity of cathelicidins

Fig. 1

**Fig. 1:**
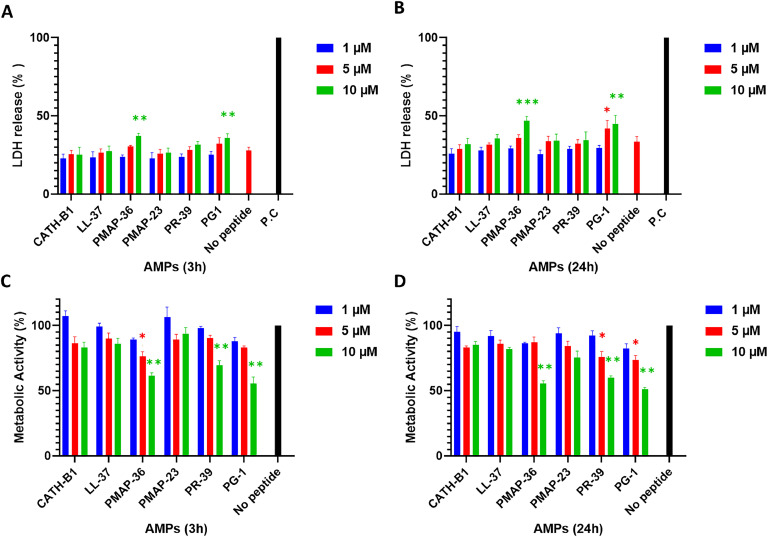
**The cytotoxicity of AMPs on Vero cells**. Cells were incubated with 0 – 10 µM AMPs for either 3 h or 24 h **A and B)** LDH release assay, indicating cell membrane permeability after 3 and 24 h, respectively. **C and D)** WST assay, measuring cell metabolic activity after 3 and 24 h, respectively. ‘No peptide‘ represents cells incubated with only growth medium for the indicated times, serving as a control. P.C in the LDH assay are cells that have been lysed by the lysis buffer representing 100% release of intracellular LDH. The reported values represent the mean ± standard deviation (SD) of three independent experiments. * *p* < 0.05; ** *p* < 0.01; *** *p* < 0.001.

**Fig. 2:**
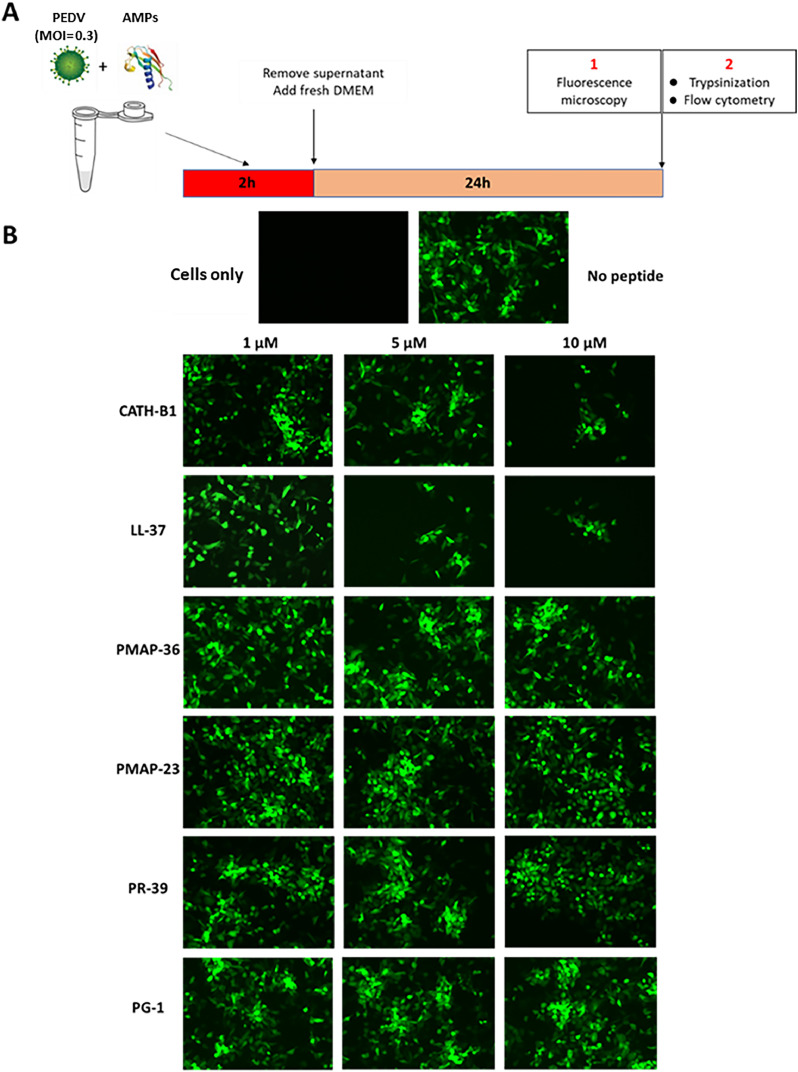
**Effect of AMPs on the infectivity of PEDV (MOI=0.3). A)** Experimental set-up of the ‘co-incubation assay’; Vero cells were subjected to 2 h exposure to a combination of AMPs and PEDV-GFP. Afterward, the supernatant was replaced, and the cells were incubated for an additional 24 h **B)** Fluorescent microscopy (20 × magnification) was performed to visualize the fluorescence signal of GFP in the presence of 1, 5, and 10 µM AMPs. The cell-only sample consists exclusively of cells while the no-peptide sample contains only PEDV-GFP, with no peptides present.

In order to determine the effect of AMPs on the infectivity of PEDV, Vero cells (5 × 10^5^ per well) were seeded in 12-well plates and cultured overnight at 37 °C, 5% CO_2_. In a co-incubation of virus and AMPs experiments (Fig. 2A), the mixture of AMPs (0–10 µM) and PEDV-GFP (MOI= 0.3) in a 1 mL volume of DMEM (P/*S* + 10% FCS) was added to the cells and incubated at 37 °C. After 2 h, the supernatant was replaced with fresh DMEM (P/*S* + 10% FCS) and incubated for 24 h. Subsequently, the cells were washed 2 times with flow cytometry buffer (500 mL PBS+ 10 mL FCS+ 250 µL of 10% NaN_3_) and were imaged with an EVOS M5000 Imaging System for semi-quantitative determination of infectivity. Eventually, cells were carefully detached by 2.5% trypsin, stained with ViaKrome 808 fixable viability dye (1:1000), and incubated for 30 min at 4 °C. Following washing, the cells were fixed using 50 µL of 4% PFA for 10 min, and a minimum of 1 × 10^5^ cells were acquired using the CytoFLEX LX Flow Cytometer (FC)(Beckman Coulter, USA). Data was analyzed using FlowJo software v.10.6 (Flowjo LCC, Ashland, OR, USA).

The effect of AMPs on viral infectivity was also determined when they were added to Vero-cells before (pre-treatment) or after (post-treatment) infection. In the pre-treatment assay (Fig. 6A), Vero cells were treated with different concentrations of CATH-B1 and LL-37 peptides (ranging from 0 to 10 µM) for various durations (1, 2, 3, 4, and 24 h) at 37 °C. Subsequently, the cells were washed 2 times with PBS and infected with PEDV-GFP at an MOI of 0.3 for 2 h

In post-incubation assays (Fig. 7A), cells were initially exposed to the virus for 2 h, washed 2times with PBS and subsequently treated with AMPs for 3 h Both for pre- and post-infection assays, after the respective incubation periods, the cells were assessed using fluorescence microscopy and quantified using FC as described above.

A larger selection of AMPs was tested in our antiviral assays to assess whether specifically porcine peptides were inactive, or if this was a general feature and the effectiveness of LL-37 and CATH-B1 would be prominent. This extended panel of AMPs encompassed chicken-derived peptides (D-CATH-2, l-CATH-2), mouse-derived peptide (CRAMP), horse-derived peptides (eCATH-1, eCATH-3), parrot-derived peptides (ER2, ER3, AG3), and synthetic peptides (CR165, CR174), all of which were readily accessible within our laboratory. As previously described, these AMPs were co-incubated with PEDV. Characteristics of these peptides are listed in table S1.

In order to determine if CATH-B1 and LL-37 physically interact with PEDV, Capto Core 700 beads (GE Healthcare Life Sciences, Chicago, IL, USA) were used to selectively eliminate unbound AMPs. PEDV were pre-incubated with or without CATH-B1/LL-37 in Opti-MEM medium, and Capto Core 700 beads were added, followed by a 1 h incubation at 4 °C while slowly rotating. After centrifugation, supernatants were collected and applied to the cells. To make sure the AMP was removed effectively, we tested samples with peptides but no virus using the same process. The collected supernatants were used to inoculate cells, and the number of infected cells was determined using the described method.

### 2.8 Transmission electron microscopy

PEDV was treated with CATH-B1, LL-37, PMAP-23, and PMAP-36 for one minute at 37 °C. Subsequently, 10 µL of the sample was placed onto a freshly carbon-coated copper grid. The grids were washed 3 times with PBS and then fixed with 1% glutaraldehyde (Sigma-Aldrich, St. Louis, MO, USA) in PBS for 10 min. Following this fixation, the grids were rinsed 2 times with PBS and 4 times with MilliQ water. They were stained for 5 min in Uranyl Acetate (pH 7) then briefly washed with methylcellulose/uranyl acetate (pH 4) and incubated for 5 min on ice. Finally, the grids were looped out of the solution and air-dried. The samples were subsequently examined in a JEM1010 (JEOL) equipped with a Veleta 2k × 2k CCD camera (EMSIS, Munster, Germany).

### 2.9 Detection of fluorogenic LL-37 by FC and confocal microscopy

To investigate the cellular localization of LL-37, Fluorogenic (FL) LL-37 was incubated with Vero cells for 3 h The cells were then washed with PBS and further incubated for 24 h at 37 °C. FC analysis was conducted to evaluate the amount of labeled LL-37 adhered or internalized within the cell, as described earlier (using unlabeled LL-37 as control). Additionally, to assess LL-37 uptake in Vero cells, the cells were incubated with FL LL-37 at concentrations of 1 and 5 µM for 30 min, 60 min, and 180 min, followed by PBS washes. After incubation, the cells were fixed with 4% PFA for 30 min at RT. After two PBS washes, DAPI (Thermo Fisher Scientific, Waltham, MA, USA) diluted in PBS (1:1000) was added for 5 min to stain the nuclei. The live cells were then imaged using a Leica TCS SP2 confocal microscope with a 63 × objective. To examine the uptake mechanism, Vero cells were first incubated at 4 °C for 30 min to inhibit energy-dependent pathways before adding FL LL-37. Subsequently, the cells were incubated with the peptide at 4 °C for 30, 60, and 180 min. FC analysis was performed to quantify the amount of FL LL-37 adhered to or internalized by the cells.

### 2.10 Statistical analysis

Statistical analysis was conducted using GraphPad Prism version 8.3.0 (538). The data were subjected to ordinary one-way ANOVA with Tukey's multiple comparison test to assess significant differences, with a significance level set at *p* < 0.05.

## 3 Results

### 3.1 Evaluation of the cytotoxic effects of AMPs on Vero cells

The AMPs employed in this study were assessed across a concentration range from 0 to 10 µM for their cytotoxicity towards Vero cells. CATH-B1, LL-37, PR-39, and PMAP-23 demonstrated no toxic effects throughout the 3 h and 24 h incubation period (Fig. 1A+*B*) even at the highest concentrations tested. In contrast, PMAP-36, and PG-1 exhibited some cytotoxicity at 5 and/or 10 µM. Similar results were observed when the metabolic activity of Vero cells was measured using the WST assay. Incubation with CATH-B1, LL-37, and PMAP-23 did not reduce the metabolic activity of Vero cells at both time points (Fig. 1C+D), while PMAP-36, PR-39, and PG-1 significantly lowered metabolic activity to 60–70% of the control.

### 3.2 *In vitro* antiviral activity of AMPs

Viral inactivation assays were conducted to assess the antiviral potential of AMPs. Six different peptides were assessed for their ability to neutralize PEDV, following the experimental protocol detailed in Fig. 2A. LL-37 was selected due to its established antiviral activity against various viruses (A Ahmed et al., 2019; Harcourt et al., 2016; Findlay et al., 2016; Matsumura et al., 2016; Alagarasu et al., 2017; Tripathi et al., 2015). CATH-B1 was also chosen based on its proven activity against IAV (Peng et al., 2020; Ye et al., 2023). Additionally, four porcine peptides were included in the study, reflecting the restricted host specificity of the virus.

**Fig. 3:**
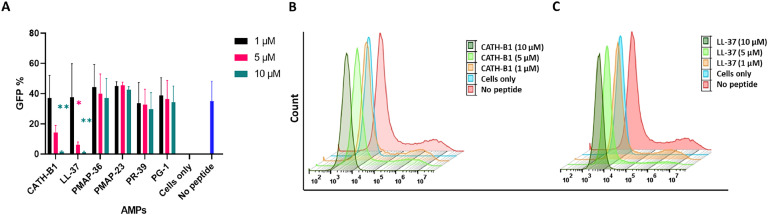
**Antiviral activity of AMPs as determined by flow cytometry (FC). A)** The percentage of GFP-positive cells following a 2 h incubation with a combination of PEDV and AMPs. **B and C)** The FC histogram shows the intensity of GFP for CATH-B1 and LL-37, respectively. The y-axis represents relative cell count, while the x-axis shows fluorescence intensity (GFP). The sample designated as 'cells only' comprises only cells, with an absence of AMPs and viruses. In contrast, the 'no peptide' sample is characterized by the presence of only the virus. The values reported here depict the average ± SD derived from three independent experiments performed in duplicate. * *p* < 0.05; ** *p* < 0.01.

Compared to the control without peptide, reduced numbers of infected cells were observed by fluorescent microscopy upon co-incubation of PEDV with 5 and 10 µM LL-37 or CATH-B1. However, the four porcine peptides did not have a clear effect on infection of Vero cells (Fig. 2B). Next, we used FC to quantify the number of GFP-positive (infected) cells. FC analysis demonstrated that CATH-B1 and LL-37 efficiently inhibited infection, leading to a significant reduction in the percentage of GFP-positive cells from approximately 35% to 1–2% at 10 µM (Fig. 3A). These results are consistent with the qualitative findings observed in fluorescent microscopy (Fig. 2B). The reduced infectivity of PEDV is also clear from the reduced total fluorescence intensity measured (histogram shifts to the left) upon incubation with either CATH-B1 (Fig. 3B) or LL-37 (Fig. 3C). On the contrary, quantitative analyses of the effect of the four other porcine AMPs on PEDV infectivity (Fig. 3A) showed no antiviral effect of these AMPs against PEDV.

**Fig. 4:**
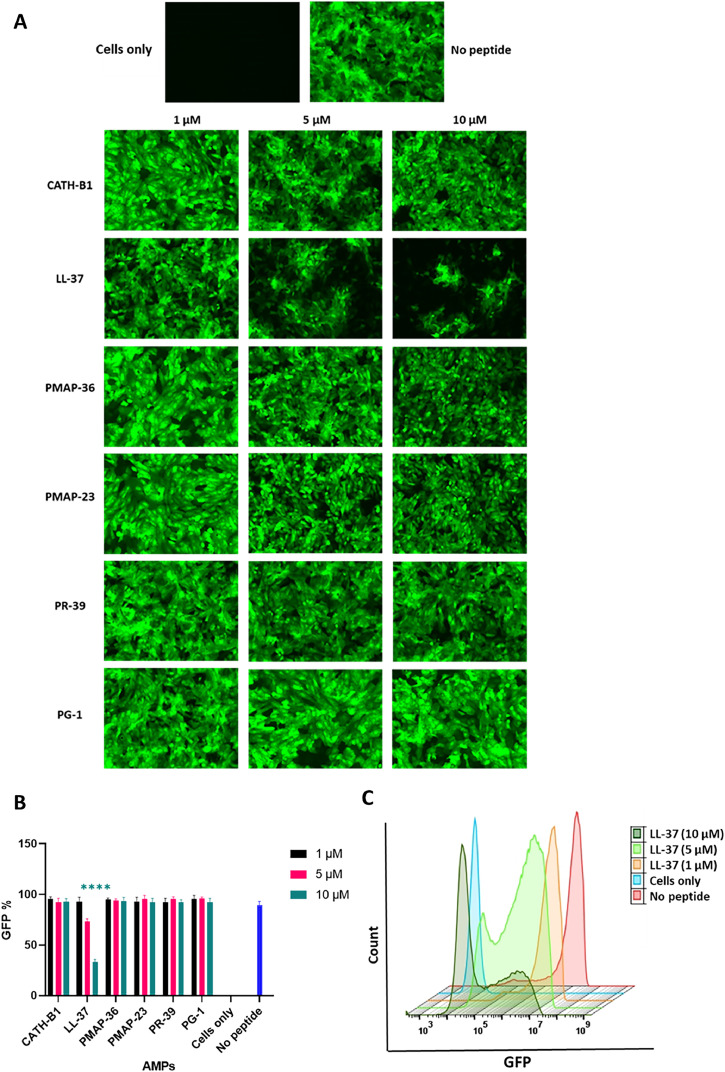
**The inhibitory effect of AMPs on PEDV at MOI=1. A)** Fluorescence microscopy image was captured at 20 × magnification to visualize the GFP fluorescence signal in Vero cells following co-incubation of AMPs and PEDV at MOI=1. **B)** The percentage of GFP-positive cells was determined by FC after 3 h co-incubation of PEDV and AMPs. **C)** The fluorescence intensity of GFP across three different concentrations of LL-37 was illustrated through the FC histogram. The y-axis represents relative cell count, while the x-axis shows fluorescence intensity (GFP). The sample designated as 'cells only' comprises only cells, with an absence of peptides and viruses. In contrast, the 'no peptide' sample is characterized by the presence of only the virus. The reported values in 4A represent the mean ± SD of three independent experiments performed in duplicate **** *p* < 0.0001.

When similar experiments were performed with higher viral levels (MOI=1) (Fig. 4), LL-37′s antiviral effect remained strong, causing a large reduction in viral infection of Vero cells at 10 µM, from almost 100 to 30 % infected cells (Fig. 4B) and a reduced total fluorescent signal (Fig. 4C). The results obtained from fluorescence microscopy further support these findings, as depicted in Fig. 4A. The antiviral impact of CATH-B1 diminished as the MOI increased, and the porcine AMPs remained ineffective as well (Fig. 4B).

**Fig. 5:**
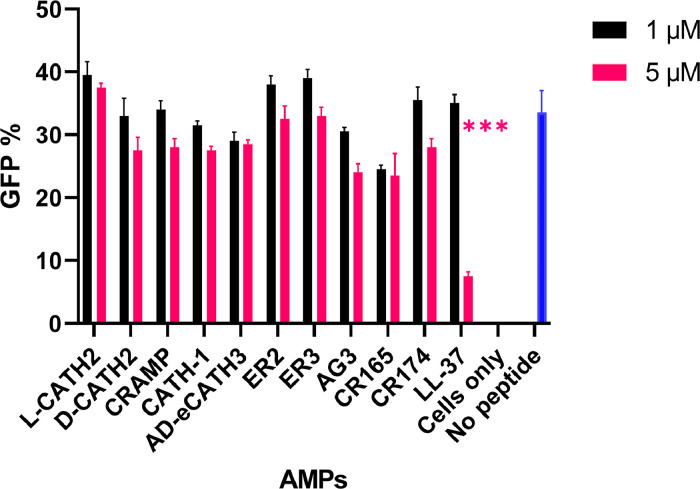
**Antiviral effects of a diverse group of AMPs on PEDV infection**. Vero cells were incubated for 3 h with a combination of PEDV-GFP and AMPs, as described previously. FC was utilized to assess the inhibitory impact of AMPs. The sample designated as 'cells only' comprises only cells, with an absence of peptides and viruses. In contrast, the 'no peptide' sample is characterized by the presence of only the virus. The values displayed represent the mean ± SD of two independent experiments in duplicate. *** *p* < 0.001.

An extended range of non-porcine AMPs was tested in the co-incubation setup to determine if the observed lack of antiviral activity could be specific to porcine AMPs. However, as shown in Fig. 5, all other tested AMPs, including an all-D isomer AMP and two non-naturally occurring synthetic (CR-165 and CR-174) AMPs were unsuccessful in significantly preventing PEDV-GFP entry into the Vero cells at a concentration of 1 and 5 µM. Again, LL-37, serving as a positive control in this set of experiments showed strong antiviral activity. These results clearly indicate that anti-PEDV activity is not related to the species origin of the AMP, but that specific characteristics of CATH-B1 and LL-37 are involved in antiviral activity.

### 3.3 Effects of AMPs on PEDV upon pre- and post-incubation

**Fig. 6:**
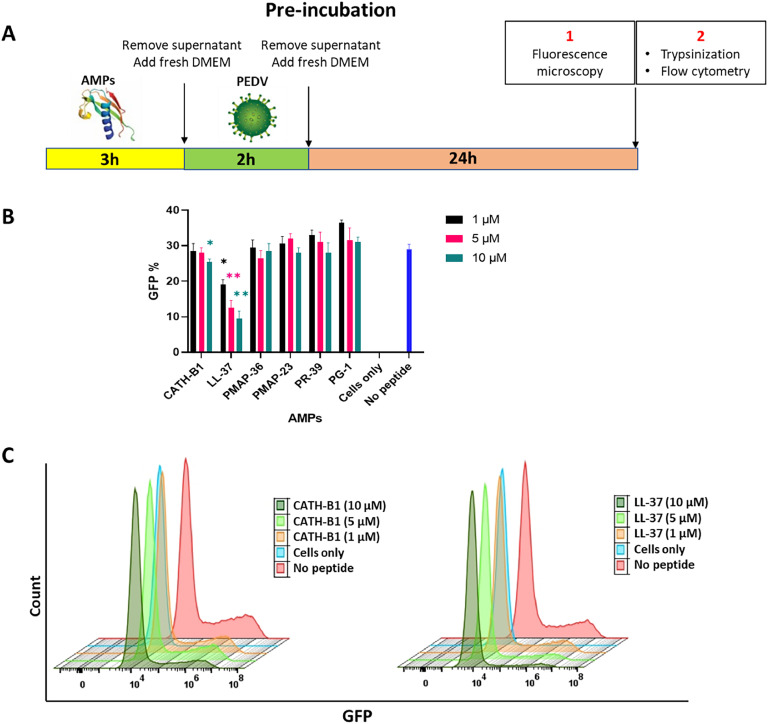
**Effects of AMPs in pre-incubation assay. A)** Experimental set-up of pre-incubation experiment. **B)** FC was employed to quantify the percentage of GFP-positive cells during pre-incubation. **C)** The histogram of FC shows the fluorescence intensity of GFP for both CATH-B1 and LL-37 during pre-treatment. The y-axis represents relative cell count, while the x-axis shows fluorescence intensity (GFP). The sample designated as ‘cells only’ comprises only cells, with an absence of peptides and viruses. In contrast, the ‘no peptide’ sample is characterized by the presence of only the virus. The reported values represent the mean ± SD derived from three independent experiments performed in triplicate. * *p* < 0.05; ** *p* < 0.01.

Initial pilot experiments indicated that LL-37 also showed antiviral activity in pre-incubation assays in which Vero cells were first incubated with the AMPs and subsequently, after removal of unbound peptide by washing of cells, were infected with PEDV. The protocol for incubating AMPs with Vero cells was optimized, and 3 h was identified as the optimal time point where the largest effect of LL-37 was observed. Subsequently, the full set of AMPs (0–10 μM) were tested for antiviral activity in this 3 h pre-incubation setup. Notably, LL-37 demonstrated potent inhibition of viral infection in Vero cells at all three concentrations, while CATH-B1 exhibited strong activity only at 10 μM (Fig. 6B, C). However, both effects were smaller than seen for co-incubation experiment. Again, none of the porcine AMPs showed a significant reduction in infected cells at these concentrations. Fluorescence microscopy qualitatively confirmed these FC results (Fig. S2).

**Fig. 7:**
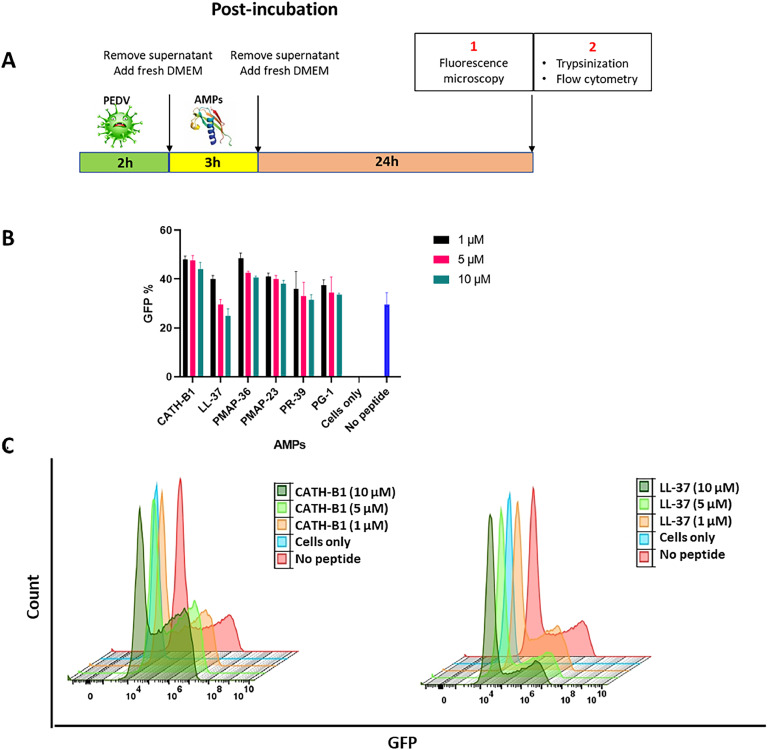
**Effects of AMPs in post-incubation assay. A)** Design and setup of post-incubation assay. **B)** The percentage of GFP-positive cells was quantified using FC during post-incubation. **C)** The FC histogram illustrates the fluorescence intensity of GFP after treatment with CATH-B1 and LL-37**.** The y-axis represents relative cell count, while the x-axis shows fluorescence intensity (GFP). The sample designated as ‘cells only’ comprises only cells, with an absence of peptides and viruses. In contrast, the ‘no peptide’ sample is characterized by the presence of only the virus. The reported values represent the mean ± SD derived from three independent experiments performed in triplicate.

Finally, the effectivity of all six AMPs was assessed when added 3 h after PEDV infection (Fig. 7B, C). The results indicate that in this ‘post-incubation setting’ AMPs are not able to reduce viral infection. Only LL-37 showed a very moderate effect in prohibiting PEDV-GFP infection into the Vero cells at 10 μM. Again, the fluorescence microscopy outcomes were consistent with the FC findings (Fig. S3).

**Fig. 8:**
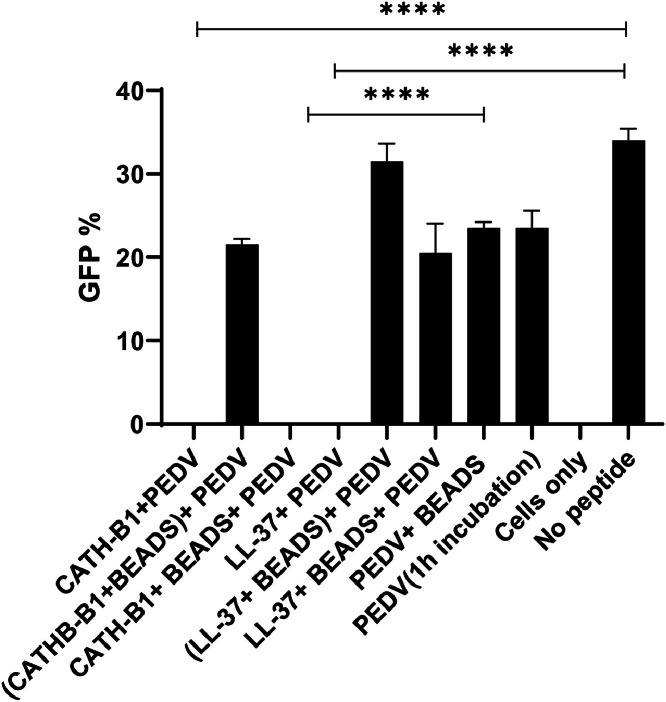
**Interactions between CATH-B1 and LL-37 with PEDV**. CATH-B1/LL-37 were pre-incubated with the virus, and subsequently, the peptide and virus were separated using Capto Core beads. The supernatant containing the separated virus was then employed to infect Vero cells. The sample designated as 'cells only' comprises only cells, with an absence of peptides and viruses. In contrast, the 'no peptide' sample is characterized by the presence of only the virus. The data is presented as the mean ± SEM of three independent experiments in duplicate, each with triplicate samples. **** *p* < 0.0001.

In order to better understand how CATH-B1 and LL-37 mechanistically block PEDV infection, the possible binding of the AMPs to the virion was studied using Capto Core 700 beads. These porous beads can bind peptides but virions are too large to enter the beads’ capillaries and therefore are not bound. When CATH-B1 and LL-37 were incubated with PEDV without beads, a decrease in virus infectivity was observed (Fig. 8), while exposing PEDV for 30 min to the beads before addition to Vero cells did not significantly change virus infectivity. Only a small reduction in infectivity was observed, which was comparable to the activity drop seen if PEDV was incubated at 4 °C without beads. This shows that the beads cannot capture the virus. Pre-incubating CATH-B1 and LL-37 with beads before PEDV introduction resulted in minimal effects on virus infectivity, suggesting an effective capture of the peptides by the beads. Interestingly, when CATH-B1 was combined with the virus and beads, its antiviral activity remained unchanged. This implies a direct interaction of the peptide with the virus, and thereby the inability of the Capto Core beads to capture the peptide in its capillaries. Conversely, LL-37 lost its inhibitory effect when combined with the virus and beads, implying a lower binding affinity of LL-37 for the virus, leading to LL-37 capturing and binding to capto core beads.

### 3.4 LL-37 and CATH-B1 affect virus morphology

**Fig. 9:**
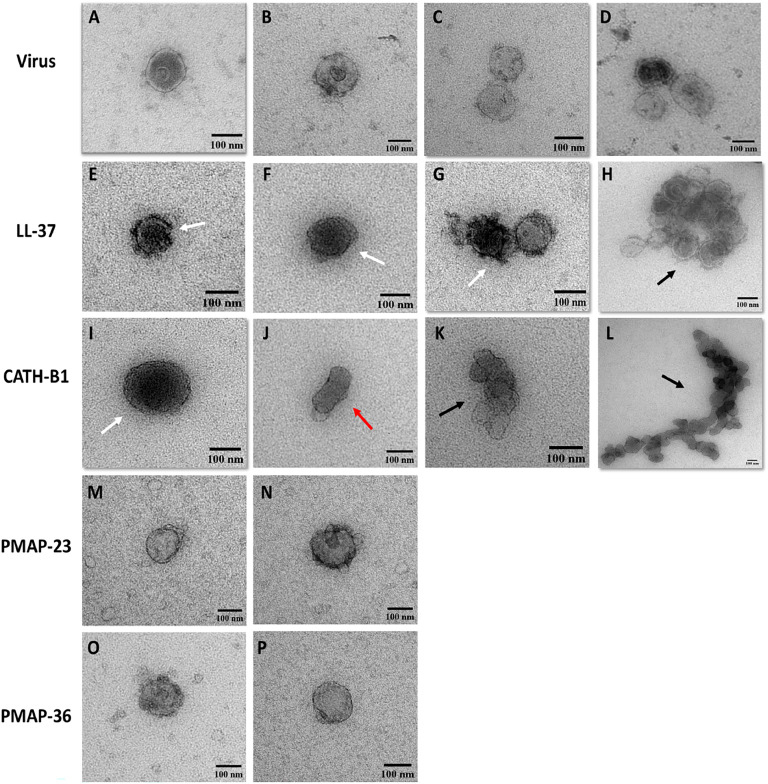
**The effect of AMPs on the morphology of the virus.** PEDV (MOI=0.3), which was pre-incubated with peptides. TEM images were taken of PEDV alone **(A–D)**, PEDV treated with 10 µM LL-37 **(E-H)**, PEDV treated with 10 µM CATH-B1 **(I-L),** PEDV treated with 10 µM PMAP-23 **(M, N**), and PEDV treated with 10 µM PMAP-36 **(O, P)**. In the images, black arrows indicate large aggregations, red arrows show deformed viruses, and white arrows highlight significant membrane damage. Micrographs were captured with a pixel size of 0,5 nm and analyzed visually using ImageJ. The micrographs are representative of observations from three independent experiments. Scale bars represent 100 nm.

To investigate how LL-37 and CATH-B1 affect the structure of viruses, we imaged negatively stained virus by Transmission electron microscopy (TEM) to determine the direct effects of LL-37 and CATH-B1 on virus morphology. Fig. 9A-D displays untreated PEDV virions, which shows the shape and size of the virus, maintaining a continuous envelope lining and sometimes showing clear spikes on the virion. There are no big aggregates of virus present (Fig. 9C, D), and generally the inside of the virion was not heavily stained, although in a small number of untreated viruses, the inside of the virion was darkly stained, likely due to viral membrane permeabilization (Fig. 9D). However, when PEDV was incubated with LL-37 (10 µM), significant membrane disruption was noted (Fig. 9E-G) and in some cases, extensive virus aggregation was observed (Fig. 9H). Similarly, incubation of PEDV with CATH-B1 (10 µM) resulted in significant membrane damage (Fig. 9I), virus deformation (Fig. 9J), and extensive virus aggregation (Fig. 9K, L). These findings suggest that direct interactions between LL-37, CATH-B1, and viral particles can damage the virion which could affect the infectivity of PEDV.

PMAP-23 (Fig. 9M, N) and PMAP-36 (Fig. O, P) were used as controls to demonstrate that the inactive peptides do not affect PEDV morphology, as the images confirmed that the virus's structure remained unchanged.

### 3.5 Uptake of LL-37 in vero cells

**Fig. 10:**
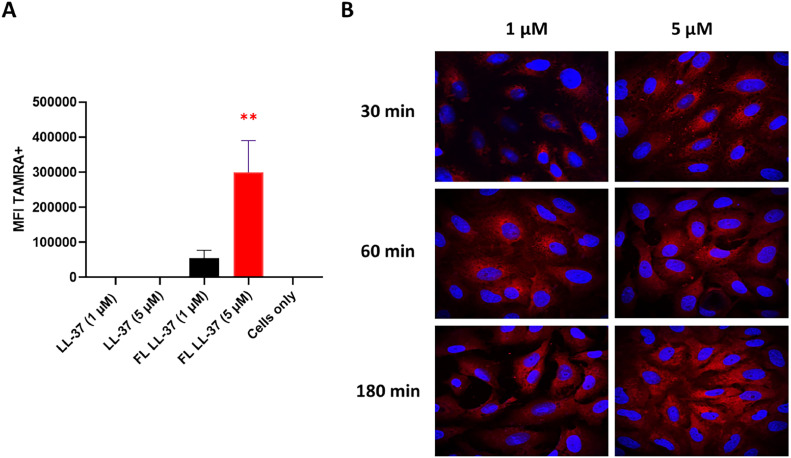
**Cellular entry of FL LL-37 into Vero cells. A)** Following a 3 h incubation with Vero cells and subsequent washing, the cellular entry of FL LL-37 and unlabeled LL-37 was examined using FC. Two concentrations of LL-37 were employed for this investigation. **B)** After treating the cells with 1 and 5 µM FL LL-37 for 30 min, 1 h, and 3 h, the cells were washed, and DAPI staining was applied before imaging using confocal microscopy. The merged image displays bright field, DAPI, and FL LL-37 signals, with nuclei shown in blue and FL LL-37 in red. The image was acquired at a magnification of 63x. The sample designated as 'cells only' comprises only cells, with an absence of peptides and viruses. In contrast, the 'no peptide' sample is characterized by the presence of only the virus. The reported values depict the mean ± SD in three different measurements in triplicate. ** *p* < 0.01.

Localization of LL-37 was determined using FL LL-37, in order to provide more insight into how LL-37 can prevent PEDV infection of Vero cells. Fluorogenic labelling of LL-37 had no effect on the antiviral activity of the peptide (Fig. S4). FC analysis, as shown in Fig. 10A demonstrated a substantial concentration-dependent uptake or attachment of FL LL-37 to Vero cells. No increase in fluorescence signal was observed for the negative control, unlabeled LL-37.

Next, confocal microscopy analysis was used to determine the cellular localization of FL LL-37 at various time points and concentrations. As shown in Fig. 10B, predominant intracellular localization of LL-37 after 1 and 3 h of incubation was observed at both concentrations. This indicates that LL-37 s antiviral activity could at least partially also be mediated by its intracellular effects on the host cell. The internalization mechanism of LL-37 was evaluated at 4 °C using flow cytometry, which showed a significant reduction in the uptake of fluorogenic LL-37, indicating that internalization occurs through endocytic pathways (Fig. S5).

## 4 Discussion

PEDV outbreaks, with high death rates among newborn piglets, have a major impact on the global swine industry. Given the ineffectiveness of current vaccines and the absence of antiviral drugs against PEDV variants, discovering new treatments is crucial (Wang et al., 2020). AMPs are known to play an important role in preventing and treating severe bacterial and viral infectious diseases by both their direct antimicrobial activity as well as their immunomodulatory activity (Lei et al., 2019; van Harten et al., 2018). Our study explored the antiviral potential of various AMPs against PEDV *in vitro*. Although LL-37 has been widely studied for its effects on multiple viruses, its efficacy against PEDV remains unexamined (Steinstraesser et al., 2005; Currie et al., 2013; Sousa et al., 2017; MD Howell et al., 2004; Ogawa et al., 2013; He et al., 2018; A Ahmed et al., 2019). Moreover, there is limited data on the antiviral activity of other peptides, highlighting the need for further investigation. In this study, besides LL-37, the efficacy of four porcine AMPs (PMAP-36, PMAP-23, PR-39, and PG-1), one chicken-derived AMP (CATH-B1), against PEDV was evaluated. Among the peptides tested, CATH-B1 and LL-37 in particular exhibited strong inhibition of PEDV in both co-incubation and pre-incubation settings *in vitro*. Somewhat surprising was the lack of activity of many other natural AMPs, considering that they contain the same general structural characteristics (cationic, helical, amphipathic). Notably, also CRAMP which has described activity against several viruses (Yu et al., 2021; MD Howell et al., 2004; Barlow et al., 2011) failed to neutralize PEDV. It is possible that the lack of anti-PEDV activity of the tested porcine AMPs partially contributes to PEDVs infectivity in pigs, although, obviously, many more factors are involved in this process.

Interestingly, based on activity reports against other viruses, LL-37 s mechanism of action can be both through direct interactions with the virus particle, but also be based on its effect on the hosts antiviral response (Pahar et al., 2020). LL-37-disrupted viral particle structures have been documented for several viruses, including DENV-2, RSV, IAV, and rhinovirus (RV) (Alagarasu et al., 2017; Currie et al., 2013; Sousa et al., 2017; Tripathi et al., 2013). Similar direct effects were also observed in this study where TEM clearly showed that LL-37 affected the structure and aggregated virus particles.

Regarding the more immunomodulatory antiviral activity, LL-37 can enter Vero cells, which could influence the host's antiviral response and viral reproduction, as shown in our pre-incubation infection experiments (Fig. 6). Several studies have shown that LL-37 can enter the endocytic pathway via binding to multiple receptors (Zhang et al., 2020; Deshpande et al., 2020; Bandholtz et al., 2006). For example in A549 epithelial cells, FPRL-1 and a second unidentified high-affinity receptor was involved in the uptake (Lau et al., 2005), while P2XR7 and CXCR2 were identified as the receptor involved in LL-37 uptake in macrophages and monocytes (Zhang et al., 2020; Tang et al., 2015). In our study LL-37 uptake was also energy dependent as incubation of Vero cells with LL-37 at 4 °C inhibited uptake, indicating that endocytosis is required.

It has been described that LL-37 stimulates immune responses by modulating cytokine and chemokine responses through activation of the hosts pattern recognition receptors. This modulation enhances IFN-β expression, thereby boosting antiviral activity against Enterovirus 71 **(**EV71) and HSV-1 infections (Yu et al., 2021; Sato et al., 2018). IFN-β initiates antiviral responses by activating key proteins such as IRF3 (Yu et al., 2021; Levy et al., 2001). However, since Vero cells are interferon-deficient and therefor do not produce Type I interferons, the reduction in infection seen in our experimental setup cannot be attributed to IFN-β production (Prescott et al., 2010; Emeny and Morgan, 1979). Similar, IFN-β independent antiviral activity in Vero cells was described for LL-37′s activity against RSV (Currie et al., 2013) and EV-71 (Yu et al., 2021) although those studies also could not depict what immunomodulatory mechanism was important. Other possibilities would include that LL-37 interferes with viral replication instead of entry as was described for HIV-1 where LL-37 specifically inhibited HIV-1 reverse transcriptase (Bergman et al., 2007; Wong et al., 2011). In addition, LL-37 can modulate (intracellular) Toll-like receptor signaling (Lande et al., 2007; Lai et al., 2011; Singh et al., 2013) can affect virus replication and in general the immune response towards viruses. This was recently described for West Nile virus (WNV) infection of keratinocytes in the presence or absence of poly (I:C) and LL-37 where both stimuli resulted in much stronger antiviral responses. Finally, a recent study showed that LL-37 bound directly to the angiotensin-converting enzyme 2 (ACE-2) binding domain, blocking this receptor for SARS-CoV-2 binding and entry into host cells (Wang et al., 2021; Lokhande et al., 2022; Li et al., 2021).

Overall, it is clear that the multitude of described immunomodulatory effects of (intracellular) LL-37 can affect the outcome of virus infections, but the exact mechanism can differ for each virus and probably even each cell type infected. Therefore, the use of Vero cells also potentially give rise to a limitation of this study, because immunomodulatory antiviral effects of (porcine) AMPs cannot be ruled out in porcine tissues or cells.

The second AMP with anti-PEDV activity analyzed in this research was CATH-B1, a chicken cathelicidin primarily found in the bursa of Fabricius, where it plays a role in enhancing the host's immune defenses against various microbial infections (Goitsuka et al., 2007). Previous research has highlighted its ability to inhibit viruses through both direct and indirect mechanisms, similar to LL-37, but only a limited number of antiviral studies have been performed with this peptide. CATH-B1 has been observed to bind to IAV particles, thereby blocking viral entry and infection (Peng et al., 2020). Similarly, *in vitro* studies have demonstrated that CATH-B1 can directly disrupt the structural integrity of pseudorabies virus (PRV) virions, thereby preventing virus binding and entry into host cells. Additionally, pre-treatment with CATH-B1 was shown to increase the antiviral immune response against PRV, leading to increased expression of IFN-β (Ye et al., 2023). The current study indicated that CATH-B1 likely exerts its anti-PEDV effects through direct interactions with viral particles. Notably, our TEM results suggest that CATH-B1 not only aggregates viruses as was seen for IAV, but also disrupts the viral structural integrity, leading to deformations in the virus morphology. The activity of CATH-B1 was lost in a pre-incubation setup indicating that at least under the tested conditions an immunomodulatory anti-PEDV role for CATH-B1 is less likely.

While peptide-based drugs show potential and are undergoing extensive clinical trials, with some already approved by the food and drug administration (FDA), challenges remain in their real-world application. These include toxicity, short lifespans and high production costs, especially for longer peptides (Agarwal and Gabrani, 2021). Therefore, in terms of drug development, using full-length CATH-B1 or LL-37 as anti-PEDV compounds in pigs may not be optimal. However, their effectiveness against pathogens and their non-toxicity to mammalian cells are crucial factors to consider, if they demonstrate both properties, they could be viable options. However, it would be beneficial to determine the structural characteristics of LL-37 and CATH-B1 that confer the antiviral activity against PEDV, and produce shorter or more (proteolytically) stable peptides. Several studies have shown that shortened LL-37 fragments can retain activity while small amino acid mutations can even increase activity (Ren et al., 2013; K Chen et al., 2021; Chingaté et al., 2015; Aghazadeh et al., 2019; Biswas et al., 2021). With the use of our developed flow-cytometry-based antiviral activity assay, this screening of LL-37-based peptides could be easily performed and would be a logical next step toward drug development.

In conclusion, our findings indicate that PEDV may possess resistance to conventional porcine AMPs, potentially contributing to its high pathogenicity. Significantly, among the peptides tested, only CATH-B1 and LL-37 demonstrated effective inhibition of PEDV *in vitro*, suggesting a direct interaction with viral particles. However, the exact mechanisms responsible for their antiviral activity remain unclear and warrant further investigation. This discovery not only enhances our understanding of the biological roles of cathelicidins *in vivo* but also underscores their potential for developing novel antiviral therapies based on this class of antimicrobial agents.

## CRediT authorship contribution statement

**Fatemeh Pashaie:** Writing – original draft, Methodology, Investigation, Data curation. **Tabitha E. Hoornweg:** Investigation, Data curation. **Floris J. Bikker:** Writing – review & editing, Supervision, Methodology. **Tineke Veenendaal:** Data curation, Methodology. **Femke Broere:** Writing – review & editing, Supervision, Funding acquisition, Conceptualization. **Edwin J.A. Veldhuizen:** Writing – review & editing, Supervision, Data curation, Conceptualization.

## Declaration of competing interest

The authors declare no competing interests.

## Data Availability

Data will be made available on request.

## Appendix Supplementary materials

Image, application 1
