# Supplementary material for: Antiviral activity of cathelicidins against porcine epidemic diarrhea virus (PEDV): Mechanisms, and efficacy
Source: Virus Res. 2024 Nov 15;350:199496. doi: 10.1016/j.virusres.2024.199496 (PMC11607671; doi:10.1016/j.virusres.2024.199496)
Supplement: Supplementary file 1 [file mmc1.docx]

**Supplementary data**

**Table S1.** **Properties of additional AMPs employed in this study.**

| AMPs | Species | Amino acid sequence | Length | Charge |
| --- | --- | --- | --- | --- |
| L-CATH-2/ D-CATH-2 | Chicken | RFGRFLRKIRRFRPKVTITIQGSARF | 26 | +9 |
| CRAMP | Mouse | ISRLAGLLRKGGEKIGEKLKKIGQKIKNFFQKLVPQPE | 38 | +10 |
| eCATH-1 | Horse | KRFGRLAKSFLRMRILLPRRKILLAS | 26 | +9 |
| eCATH-3 | Horse | KRFHSVGSLIQRHQQMIRDKSEATRHGIRIITRPKLLLAS | 40 | +9 |
| CR165 | Designed | RRWVQRWIRWVQRWVR | 16 | +6 |
| CR174 | Designed | RRWVQRWIRRWRKVAAARRWVQRWIRRWRPKV | 32 | +14 |
| ER2 | Parrot | LVQRGRFGRFLGKIRRFRPKVKFYAKAGVSVSLG | 34 | +10 |
| ER3 | Parrot | RVKRFWPLLVKAIKTVATGVGIFKSFKG | 28 | +7 |
| AG3 | Parrot | RVKRFWPLLVTAIRTVAAGVGIFKSFKG | 28 | +6 |

**
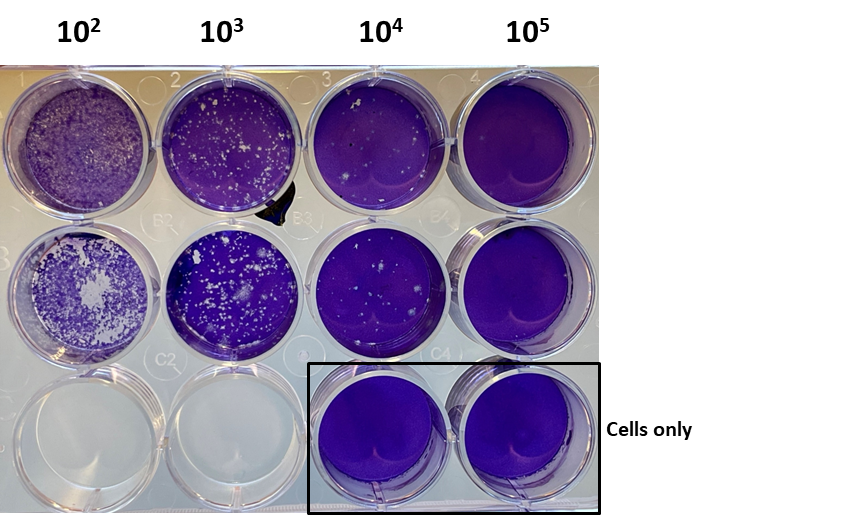
**

**Figure S1.** **Viral titers were assessed using a plaque assay.** Clear regions within the blue cell monolayer, known as plaques, indicate areas of virus infection. The results of plaque reduction assay were observed, with the progressively diluted virus samples (10-fold dilutions) being added to the cells and subsequently incubated at 37 °C for 2 h. The 'cells only' wells include cells alone, with no presence of virus.

**
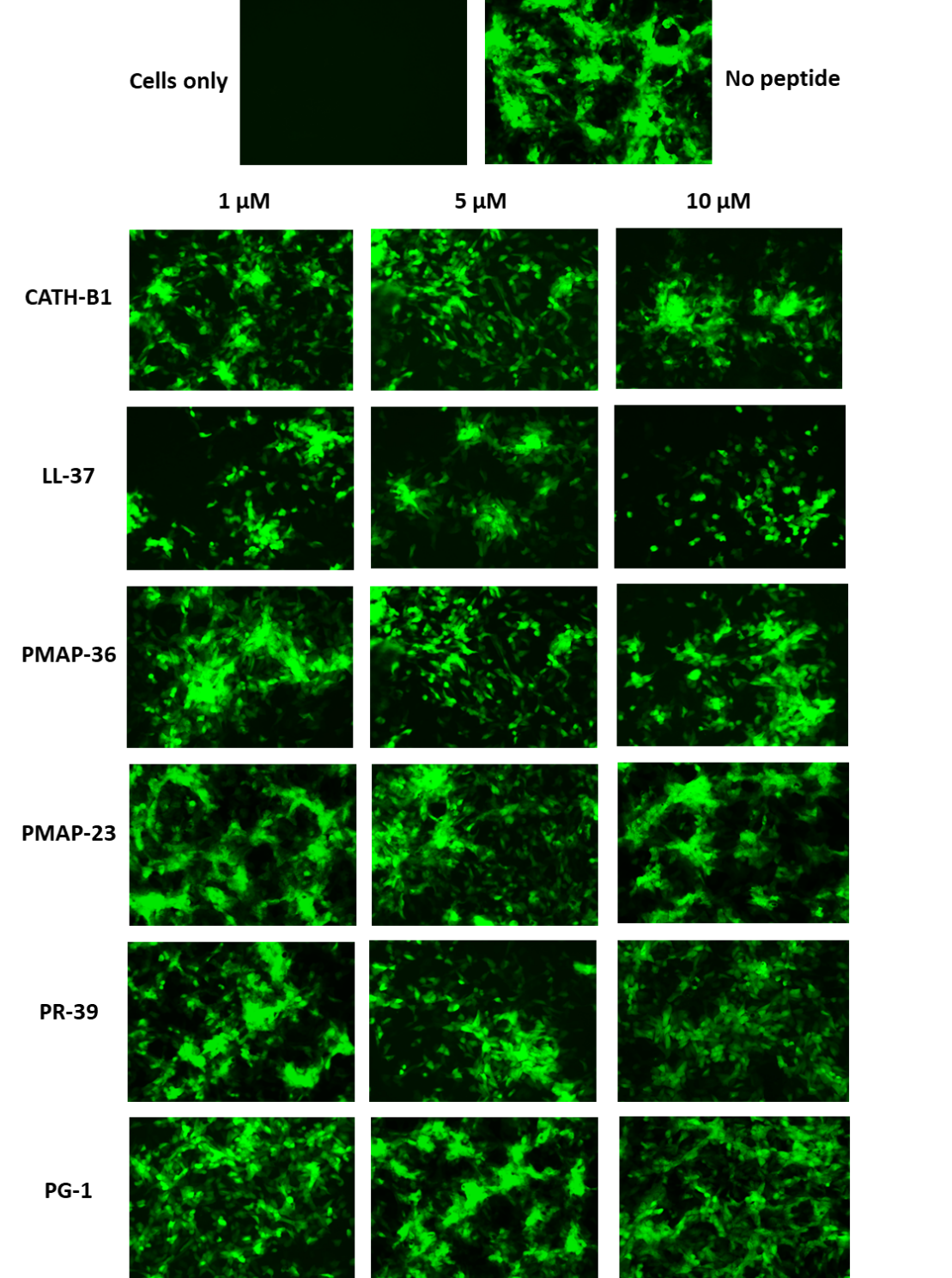
**

**Figure S2**. **Fluorescent microscopy analyses of PEDV-infected Vero-cells in pre-incubation with AMPs** (at a magnification of 20x). The sample designated as 'cells only' comprises only cells, with an absence of peptides and viruses. In contrast, the 'no peptide' sample is characterized by the presence of only the virus.

**
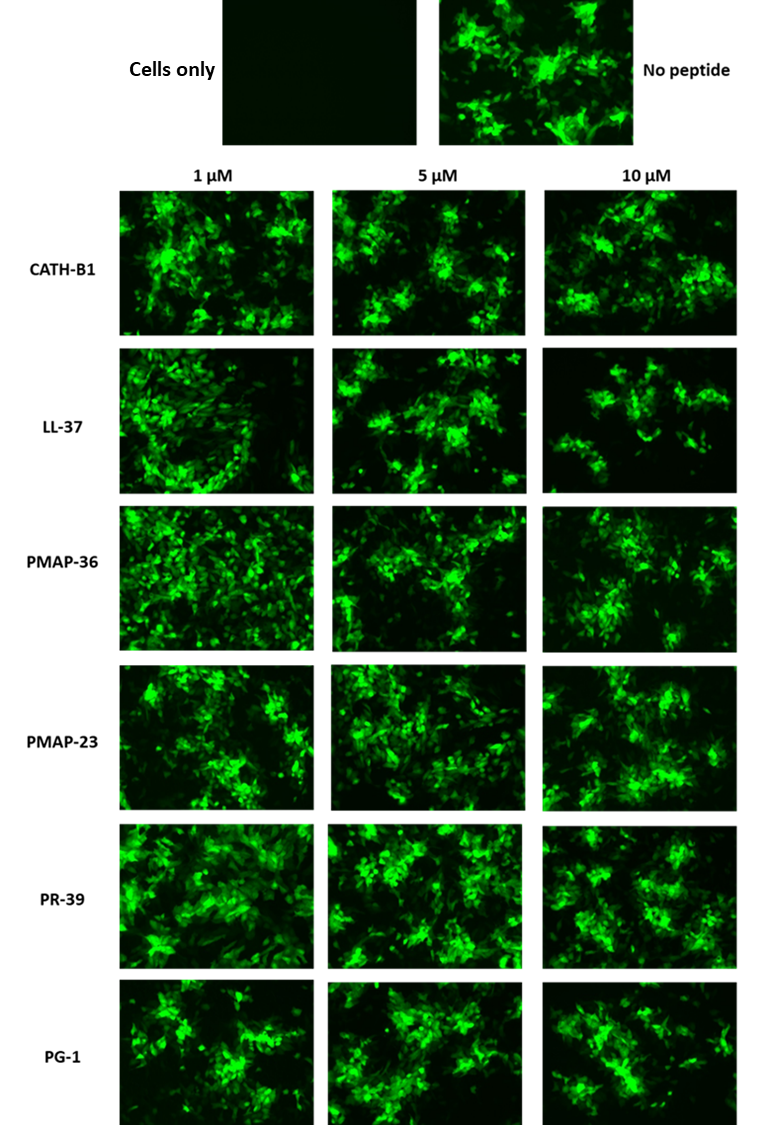
**

**Figure S3**. **Fluorescence microscopy image was utilized** **at a magnification of 20× to visualize the GFP fluorescence signal in Vero-cells during AMPs post-treatment**. The sample designated as 'cells only' comprises only cells, with an absence of peptides and viruses. In contrast, the 'no peptide' sample is characterized by the presence of only the virus.

**Figure S4.** **The antiviral activity of FL LL-37 (fluorogenic LL-37 ) and unlabeled LL-37**. Peptides were co-incubated at 1 and 5 µM with PEDV and added to Vero cells for 3 h, followed by FC analysis to examine the infectivity. The sample designated as 'cells only' comprises only cells, with an absence of peptides and viruses. In contrast, the 'no peptide' sample is characterized by the presence of only the virus. .* p < 0.05; *** p < 0.001; **** p < 0.0001.


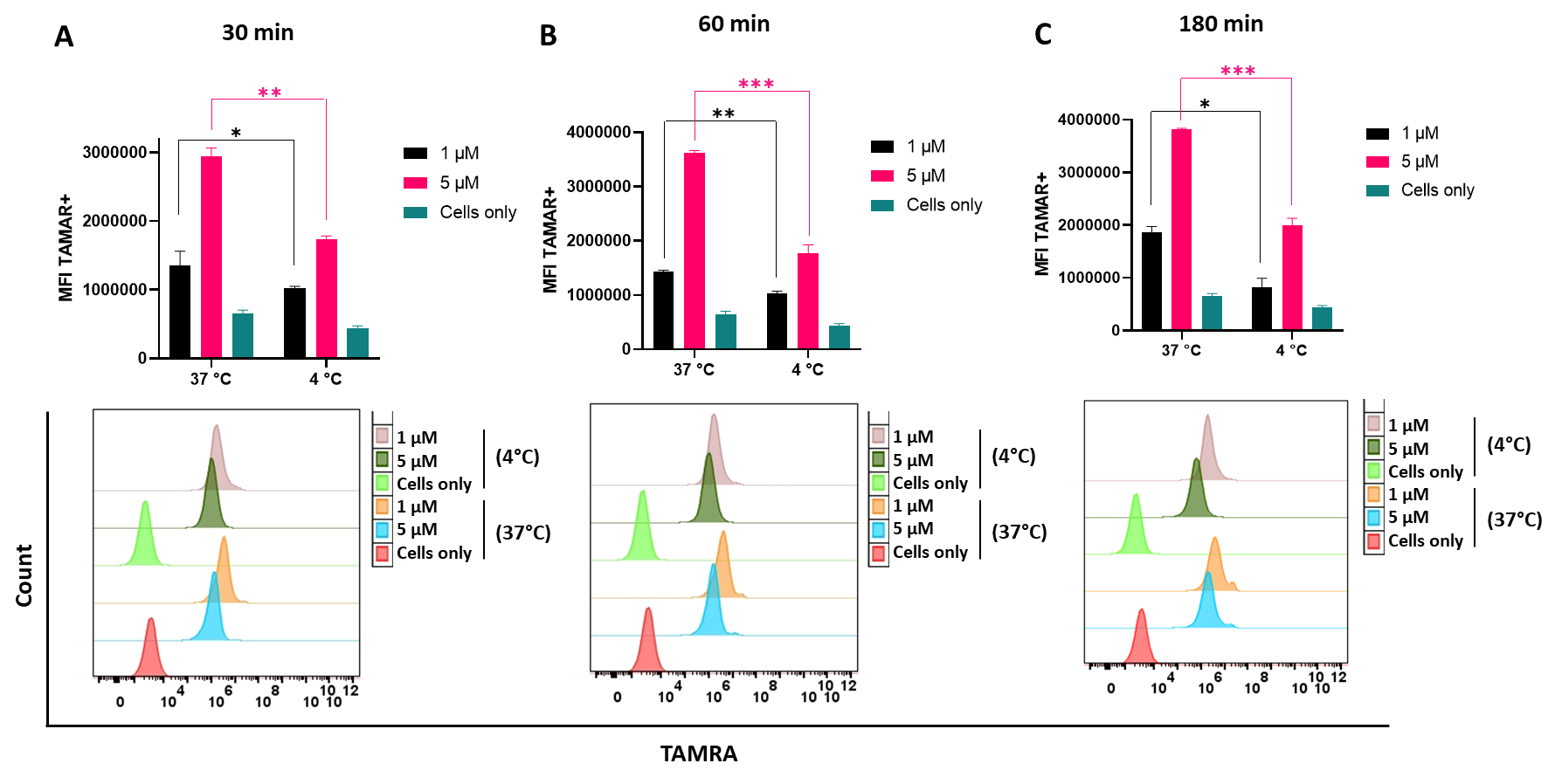
**Figure S5.** Cellular uptake of fluorescein-labeled LL-37 (1 μM and 5 μM) in Vero cells was assessed after incubation for **A)** 30 min, **B)** 60 min, and **C)** 180 min, using a temperature-controlled assay at both 37 °C and 4 °C. Untreated cells (cells only) were used as the control. FC was employed for analysis..* p < 0.05; ** p < 0.01; *** p < 0.001.
